# Mycobiota community and fungal species response to development stage and fire blight disease in apples

**DOI:** 10.3934/microbiol.2023029

**Published:** 2023-07-20

**Authors:** Su In Lee, Gyeongjun Cho, Su-Hyeon Kim, Da-Ran Kim, Youn-Sig Kwak

**Affiliations:** 1 Division of Applied Life Science (BK21 Plus), Gyeongsang National University, Jinju 52828, Republic of Korea; 2 Division of Agricultural Microbiology, National Institute of Agriculture Science, Rural Development Administration, Wanju 55365, Republic of Korea; 3 Research Institute of Life Science, Gyeongsang National University, Jinju 52828, Republic of Korea

**Keywords:** *Erwiana amylovora*, fungal community, fruit crops, *Lipomyces*, microbiome

## Abstract

Fire blight disease, caused by the bacterial pathogen *Erwinia amylovora*, has been a significant concern for over 50 countries worldwide. The efficacy of chemical pesticides currently available for disease control is limited. To address this issue, research is being conducted to explore environmentally friendly control methods, particularly biological control using beneficial microorganisms. However, there is limited research on the apple microbiota community and minimal research has been conducted on fungal communities that may exhibit reliable performance in apple trees. Therefore, our objective was to analyze the fungal communities present in apples at different developmental stages and in different tissues, aiming to identify potential biological control agents for fire blight disease. Our findings indicate that the fungal communities present in apple buds, flowers and leaves play an important role in inhibiting the invasion of *E. amylovora*. Specifically, we propose GS11 and *Lipomyces starkeyi* as potential keystone taxa that respond to fire blight disease. These findings provide insights into the continuity and discontinuity of fungal community structure in different developmental stages of apples and offer predictions for potential biological control agents for fire blight disease.

## 1. Introduction

Apples are widely grown all over the world and are economically important fruits. The production of apples is constantly increasing and the area of cultivation is also expanding [1]. Among agricultural products, apples occupy a very important position nutritionally and are known as a healthy fruit for humans as they contain various polyphenolic compounds and vitamins [2]. Various diseases occur during apple cultivation and most major diseases are known to be caused by fungi. The main diseases that cause significant damage to apple cultivation and production include white rot, scab, powdery mildew, leaf spot and anthracnose [3],[4]. To prevent apple diseases, a minimum of 10 pesticide applications are used in apple orchards [5].

Fire blight disease, caused by the Gram-negative bacteria *Erwinia amylovora*, has been causing severe damage to apple production worldwide since it first appeared in North America in 1780 [6]. The disease has been reported in about 50 countries around the world. East Asia, including South Korea, had been a region where the fire blight disease had not been reported until recently. However, since the outbreak of fire blight disease in South Korea in 2015 many orchards have shown the damage caused by the disease [7]. The primary mode of transmission of the fire blight pathogen in apple trees is well-established to be through pollinators with bees being particularly significant. The fire blight bacteria exhibit the possession of two copies of the type III secretion systems with one copy playing an indispensable role in pathogenicity while the other potentially facilitates their survival within the gut of pollinators [8]. Once the bacteria infect the floral tissue they can then move to the developing fruit and begin to colonize and reproduce, leading to symptoms on young twigs and leaves such as die-back and blackened symptoms. As the disease progresses, it can cause defoliation and cankers on the branches [3],[9]. Due to the perennial nature of apple trees, *E. amylovora* can continue to cause damage for multiple growing seasons and during the early spring cankers can exude a sticky liquid called ooze that attracts insects, increasing the potential for the spread of the bacteria to other apple trees during the flowering period [10],[11].

Antibiotics such as streptomycin and oxytetracycline as well as pesticides such as oxolinic acid and copper-based antibacterial chemicals are being used for the treatment of the fire blight disease [12],[13]. However, the effectiveness of antibiotics and chemical pesticides in controlling and managing the disease is not clear and there are concerns about the emergence of antibiotic-resistant pathogenic bacteria and toxicity within a relatively short period of time [14],[15]. Recently, biological control has been gaining attention as a means for maintaining ecosystem stability and sustainable agriculture. The foundation of plant disease biological control was established through the discovery of the colonization and disease-suppressive capabilities of *Pseudomonas* in natural suppression soil of the take-all disease in wheat fields [16],[17]. Subsequently, various research studies have been conducted on the utilization of biological control agents for the prevention and management of crop diseases. Studies on the biological control of fire blight in apples are also being conducted by various research teams. Microorganisms known to be as beneficial agents for plants such as *Bacillus* spp.*, Lactobacillus plantarum, Pantoea agglomerans* and *Aureobasidium* spp. which belong to the Basidiomycota fungi have been investigated for their effects and mechanisms in controlling the fire blight in apple and pear [18]–[20]. However, the effectiveness of biological control utilizing beneficial microorganisms is not significantly higher compared to traditional chemical pesticides and there is a lack of visible efficacy in disease control. This is because many biological control agents are evaluated and obtained under in vitro conditions. The inability to effectively identify an agent for disease suppression through the ecological and evolutionary mechanism of mutualism or symbiosis between particular crops and organisms with a strong affinity despite an extended duration of co-evolution represents a significant shortcoming [21].

These limitations can be overcome through the analysis of NGS-based plant microbiota which can provide biologically and evolutionarily relevant potential biocontrol agents based on interactions with the target crops. Kim et al. [22] conducted a comparative analysis of the microbiota community structure in relation to the infection status of apple fire blight in various plant tissues such as the root, fruit, leaf and shoot. Interestingly, the microbial community structure in the root showed high microbial diversity regardless of fire blight infection. Moreover, there were no statistically significant differences in microbial community structures among the clusters. The leaf samples also revealed no changes in microbial community structure due to fire blight infection. However, the fruit and shoot samples displayed significant structural changes in their microbial communities in response to fire blight infection. Disease-free apple samples displayed active metabolic pathways and 13 of them were found to be statistically highly expressed only in the microbial community of disease-free apple samples [22]. According to a recent paper that compares the microbial communities of apple flowers in relation to the occurrence of fire blight, the dominant microorganisms in disease-free apple flowers were found to be *Pantoea agglomerans* and *P. allii* [23] In fire blight diseased flowers, the microbial community was observed to be dominated by *E. amylovora* in over 90% of the cases. Interestingly, a comparison of gene expression between disease-free and fire blight diseased flowers revealed that glucose and xylose metabolism were highly active in flowers infected with fire blight [23].

In various fruit crops, fungi are recognized as potential biological control agents. However, the research on fungi communities using NGS for the control of fire blight disease and the selection of beneficial fungi candidates is limited. Therefore, this study aimed to analyze the fungi communities in apple tissues, roots, stems, leaves and fruits at different developmental stages and the fire blight disease occurrence in order to identify the fungal species related to the suppression of fire blight outbreaks.

## 2. Materials and methods

### 2.1. Sampling description

Apple (*Malus domestica* cv. Fuji, tree age 20 to 25 years old) organizations were sampled five times. The first to fourth samplings were performed in a disease-free orchard and the fifth sampling was performed in both the fire blight diseased orchards and the disease-free orchard (Table 1). The sampled organization was rhizosphere (1st~5th), flowers (2nd~3rd), leaves (1st, 4th and 5th sampling), freshly grown twigs (4th~5th) and pre-mature fruits (5th) from designated trees (n = 5). Rhizosphere soil (filled in a 50 mL tube) was obtained from 1 to 3 mm near the surface of fine roots. The flowers were plucked by about 1 g in the budding stage (4th) and flowering stage (5th). The leaves were picked by about 2 g in the budding stage (1st) and about 5 g in the expansion stage (4th~5th). The twigs were cut by about 1.5 g. Two to three fruits were harvested from each tree. These samples were packed in a plastic bag, stored prechilled iced box and transported to a deep freezer (–80 °C) in the laboratory.

### 2.2. Microbial DNA extraction

The collected samples (except rhizosphere soil) were washed by sonication (35 kHz for 10 min) with pH 7.4 PBS buffer (80 g, NaCl, 2 g KCl, 1.44 g Na_2_HPO_4_ and 2.4 g KH_2_PO_4_ in 1 L of autoclaved distilled water) and the sonicated PBS buffer was centrifugated for collect fungi living in episphere of fruit and leaves. To isolate endosphere fungi, the tissue samples were disinfected by 70% ethyl alcohol for 30 sec and 1% sodium hypochlorite solution for 30 sec. The solution was rinsed twice with autoclaved distilled water. To extract metagenomic DNA, 500 mg of the rhizosphere, 300 mg of endosphere samples, and episphere pellet were processed that there were homogenized with glass beads by FastPrep instrument which set 6.0 speed for 40 sec, protein in the samples were precipitated and removed and DNA was collected using DNA-binding matrix in FastDNA ^®^ SPIN kit for soil (MP Biomedicals, Irvine, CA) kit.

**Table 1.** Sampling information.

Sampling time number	Date	Orchard (GPS)	Organization
1	2020.03.09	Disease-free orchard (36.9, 127.6)	rhizosphere, leaves (budding stage)
2	2020.04.08		rhizosphere, flower (budding stage)
3	2020.04.21		rhizosphere, flower (flowering stage)
4	2020.05.14		rhizosphere, leaves (expansion stage), freshly grown twigs
5	2020.06.01		rhizosphere, freshly grown twigs, fruits
5	2020.06.03	Fire blight diseased orchard A (36.9, 127.7)	rhizosphere, freshly grown twigs, fruits
5	2020.06.03	Fire blight diseased orchard B (37.0, 127.7)	rhizosphere, freshly grown twigs, fruits

### 2.3. ITS amplified library construnction

ITS2 region was amplified by MiSeq adapter linked to the forward primer, 5.8S-Fun (5′TCGTCGGCAGCGTCAGATGTGTATAAGAGACAGAACTTTYRRCAAYGGATCWCT, 1 µM) and the reverse primer, ITS4-Fun (5′GTCTCGTGGGCTCGGAGATGTGTATAAGAGACAGAGCCTCCGCTTATTGATATGCTTAART, 1 µM). Each primer (5 µL), the DNA template (1 µL), H_2_O (1.5 µL) and 2 x KAPA HiFi HotStart ReadyMix (Roche, Basel, Swiss). Its thermal condition was 95°C in initial denaturation and denaturation, 55 °C in annealing and 72 °C in synthesis and final synthesis. The cycle composited by denaturation for 30 sec, annealing for 30 sec, and synthesis for 30 sec was 27 in the endosphere and 30 in the rhizosphere and episphere. The initial and final steps were three min and five min. The amplifications were purified by using AMPure XP (Beckman Coulter, USA) which were DNA clean up kit based on magnetic bead.

### 2.4. Fungal microbiota community analysis

Illumina MiSeq of the library was entrusted by Macrogen cooperation (Seoul. Korea). Phred quality score included sequence was received by fastq format files. The primer sequence was removed by Cutadapt (version 2.9) and reads containing N, meaning any sequence, were removed by the dada2 package (version 1.16) in R (version 4.0.3) The filtered reads were machine-learned error using phred score and clustered using the dada2, ASV clustering program based on divisive amplicon denoising algorithm. The clustered ASVs were identified by naive bayes classifier method with UNITE database (version 8.3), fungal ITS region database. Alpha and beta diversity were analyzed by the vegan (version 2.5-0) R package. Erwiniaceae relative abundance was referred to the previous studies [22] because the ITS library was amplified by the same metagenomic DNA. Distribution analysis of fungi is performed by analysis of compositions of microbiomes with bias correction (ANCOM-BC). The fungal functional analysis was performed by FunGuild (version 1.1).

**Figure 1.**
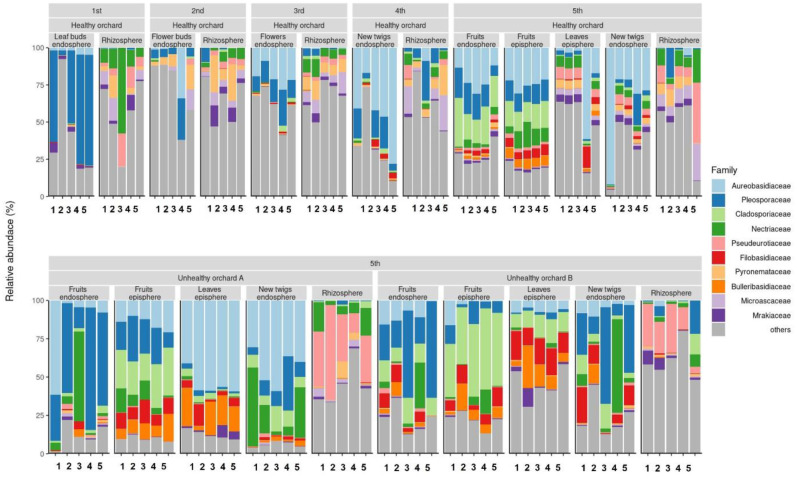
Relative abundance of taxonomic families. The y-axis represents the mean relative abundance, and the color corresponds to the taxonomic group at the family level. Samples collected under the same conditions (sampling time, orchard and organization) are plotted along the horizontal axis.

## 3. Results and Discussion

### 3.1. Fungi abundance is affected by apple development stages and fire blight disease

To reveal the fungi community structure present in different developmental stages of apples, samples were collected from the rhizosphere, buds, flowers, leaves and fruits at different development stages. Additionally, the samples were also collected from two apple orchards where the fire blight disease occurrence was confirmed in early June, the period when the symptoms were most obvious, to investigate the shifting in fungi community structure based on the occurrence of fire blight disease (Table 1). The result of the rarefaction curve which was used to confirm whether the obtained NGS read number was sufficient for analyzing fungi community structure showed that the quantity and quality of the obtained nucleotide sequences were both satisfactory for further studies. The lowest number of amplicon sequence variants (ASV) was observed in the flower bud and new twig endosphere. Rhizosphere showed the highest fungal ASV number among the samples (Figure S1). The result of analyzing the relative abundance of fungi communities in relation to the developmental stages of apples indicated that little variation was observed in the rhizosphere from the onset of buds to fruit maturity stages. The high relative abundance of fungi belonging to the families Nectriaceae, Pleosporaceae and Pyronemataceae was observed (Figure 1). However, a difference in the abundance of fungi communities in the rhizosphere of orchards affected by the fire blight disease was observed compared to the disease-free orchard. In the fire blight occurred apple rhizosphere, Pseudeurotiaceae was the highest abundance of fungi in both fire blight disease orchards (Figure 1). The abundance of fungi at different development stages of disease-free apples revealed that at the leaf bud (stage 1) period, Pleosporaceae was the dominant taxa and during the flower bud (stage 2 both Pleosporaceae and Pyronemataceae were confirmed as relatively abundant fungi in the community. Interestingly, during the flower bud (stage 3) Aureobasidiaceae was the most abundant fungi. Furthermore, during stages 4 and 5 which correspond to the new twig and the fruit stages the abundance of Aureobasidiaceae further increased in the apple endosphere (Figure 1). Aureobasidiaceae is known to be an excellent biological control agent in the management of various fruit diseases. This is speculated to be because fruits store large amounts of carbon nutrients such as fructose and glucose which are making favorable conditions for Aureobasidiaceae to colonize on the fruit surface or in the endosphere [24]. Recent studies have also shown that the yeast species belonging to the Basidiomycetes are being evaluated as potential biocontrol agents for the fire blight disease in apples and pears, providing a scientific foundation for their trial [25],[26].

### 3.2. Different fungi diversity by E. amylovora infection

**Figure 2.**
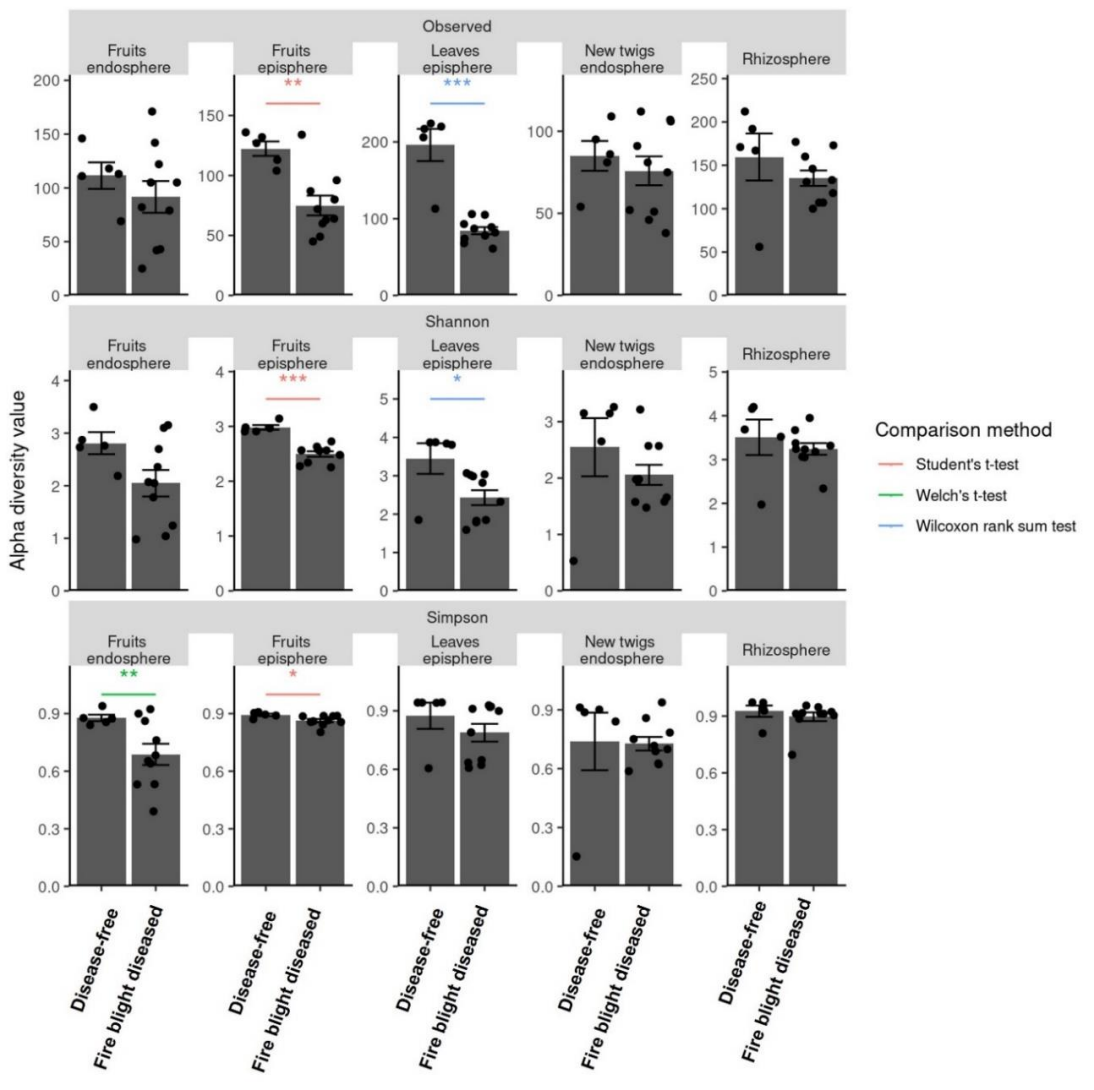
Alpha diversity comparison between disease-free and fire blight diseased trees at the 5th sampling timepoint. Each comparison was subjected to appropriate statistical tests to ensure normality and equal variance. When normality was not met, the Wilcoxon rank sum test was used. The student-T test was employed when normality and equal variance were met and the Welch's-T test was used in cases where normality and equal variance were not met. Significance levels are indicated as follows: (*: *P* < 0.05, **: *P* < 0.01, ***: *P* < 0.001).

Alpha diversity among different tissues within the same development stage of apple was highest in the rhizosphere (Figure S2A). In apple tissues collected at different development stages, alpha diversity of fungi in samples other than the rhizosphere showed similar diversity values. Interestingly, a similar level of fungi alpha diversities was also observed among apple tissues even in fire blight diseased orchards which were infected with the fire blight pathogen (Figure S2B,C). The results of comparing the fungi community alpha diversity value of each apple tissue according to the incidence of fire blight showed that the observed diversity value was higher in the disease-free leaves (Figure 2). However, when using the Shannon index a statistically significant difference was revealed in both the disease-free apple fruits episphere and leaves episphere. Interestingly, the results according to the Simpson algorithm also showed a high diversity value in both the fruits endosphere and the fruit episphere in the disease-free orchards (Figure 2). Despite the onset of fire blight, the diversity of fungi community in the rhizosphere remained consistent. However, the fungi diversity in the episphere and endosphere of fruit and leaves decreased by *E. amylovora* infection. The findings suggest a potential correlation between the occurrence of fire blight disease and microbiota. Furthermore, it is shown that selecting candidate microorganisms that can control *E. amylovora* among the species present in the disease-free apple leaves and fruits is a more appropriate approach because it targets the specific microbial population present in the orchard ecosystem.

### 3.3. Fungal community groups correlated with apple tissue

**Figure 3.**
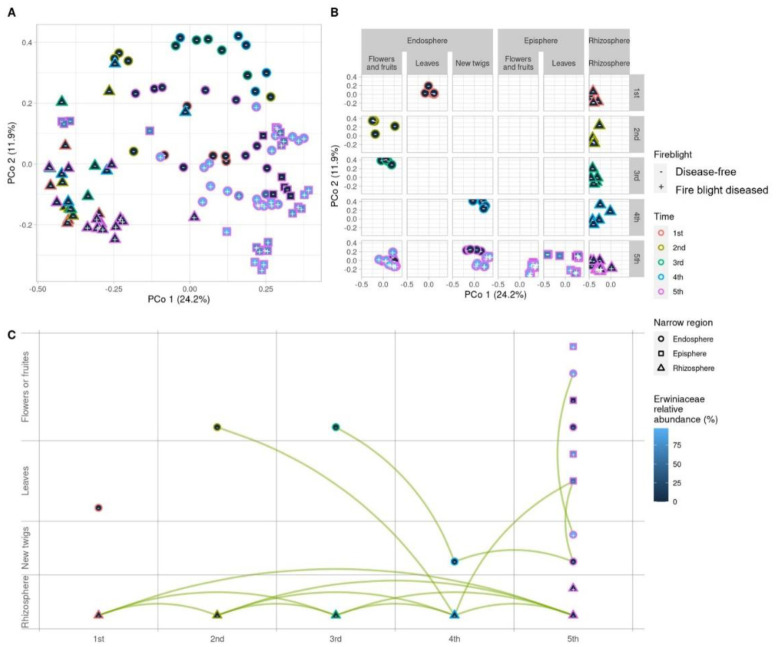
Beta diversity analysis based on Bray-Curtis distance of ASV relative abundance. (A) The classical multi-dimensional scaling technique, principal coordinate analysis (PCoA), is used to represent the Bray-Curtis distance in a planar graph. (B) The planar graph is divided by sampling time and organization with the edge color and spot shape indicating the sampling time and organization, respectively. The filled color inside the spot represents the relative abundance of Erwiniaceae in previous studies while the plus and minus signs inside the spot denote disease-free and fire blight diseased trees, respectively. (C) The network is simplified using pairwise PERMANOVA analysis which is adjusted by false discovery rate. The green line connects two sample groups that are statistically indistinguishable (*P*_adj_ > 0.05).

The Bray-Curtis distance analyses revealed that the beta diversity of the fungal community in apple trees is most similar by a factor of tissues among the different developmental stages, tissues and whether fire blight infection or not (Figure 3A and Table S1). Further analysis of the beta diversity of the fungal community in relation to developmental stages, tissues and fire blight infection status showed that only in the endosphere of twigs was there a distinction between the beta diversity of disease-free and fire blight diseased orchards (Figure 3B). As determined by the alpha diversity results, the fungal community in the rhizosphere formed a single group in PCoA regardless of the presence of fire blight disease. This suggests that the composition of fungal communities in the rhizosphere is not significantly influenced by the incidence of fire blight disease. We utilized the non-parametric statistical test permutational multivariate analysis of variance (PERMNOVA) to analyze the network's centroid and dispersion of the fungal communities (Figure 3C). The results of the analysis indicated that the fungal community resent in the rhizosphere were all similar during apple development stages (1^st^–5^th^). Furthermore, the fungal community in the bud of flowers and fruits was found to be like those in the 4^th^ stage rhizosphere and 5^th^ stage leave samples. The observation of the centroid of community in the flower and fruit of the 3^rd^ growth stage and the twigs of the 4^th^ and 5^th^ growth stages revealed similarities. Interestingly, in the 5^th^ growth stage of the apple the similarity in the centroids was observed to form among different tissues (fruits, leave and twigs) regardless of whether the communities present in the endosphere or episphere.

**Figure 4.**
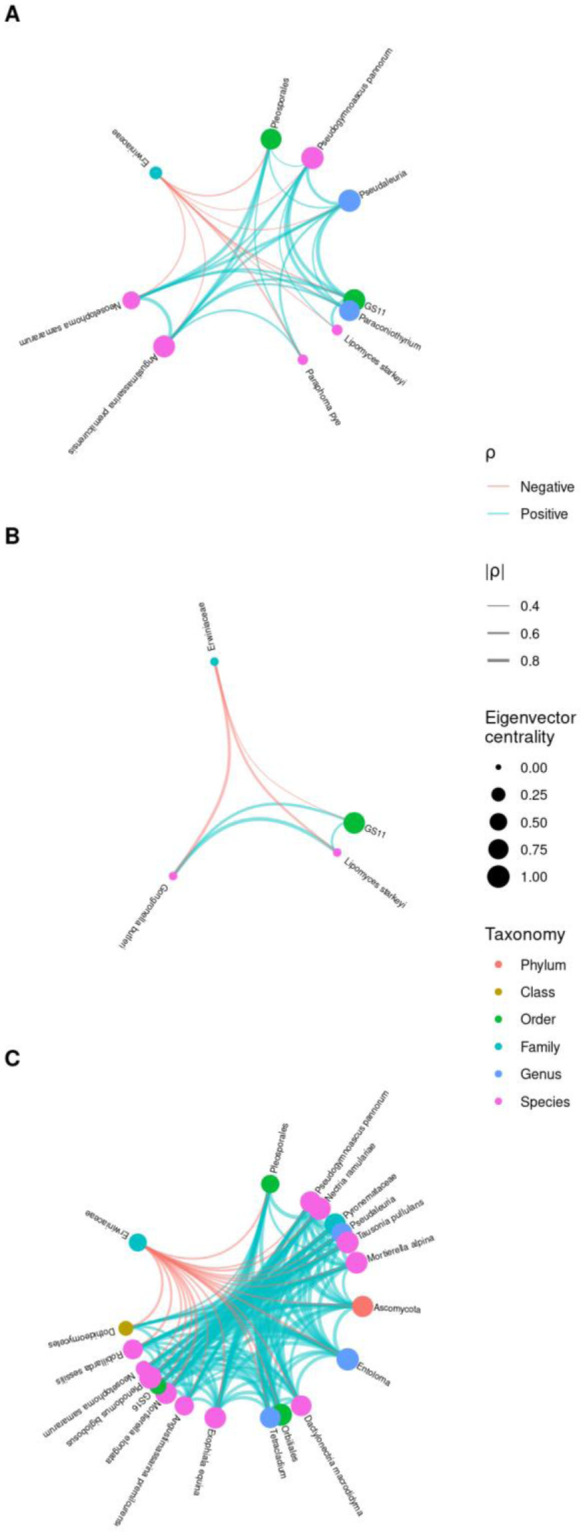
Pearson correlation network between Erwiniaceae and fungi in all sampling times. (A) includes all endosphere and episphere samples. (B) includes only endosphere samples. (C) includes only episphere samples. The Pearson correlation values are adjusted using the false discovery rate method. The network displays the correlation results of Erwiniaceae-associated fungi with node size and color indicating taxonomy levels and eigenvector centrality among fungi and Erwiniaceae. Line thickness and color indicate the magnitude of the correlation. (*P*_adj_ < 0.01).

The PERMANOVA results showed similar trends to those obtained in the alpha and beta diversities results, indicating that the fungal communities in the rhizosphere were not affected by the incidence of fire blight and retained a similar community structure. As reported by Kim et al. [22], the correlation between the bacterial microbiota community structure in apples and the incidence of fire blight also suggested that the bacterial community structure in the apple rhizosphere is not affected by the fire blight disease. These results imply that the soil microbiota may not be a major target for managing apple fire blight disease. The findings suggested that there is no need to indiscriminately apply antimicrobial pesticides throughout the orchard for the purpose of fire blight control. The association of centroid in the 2^nd^ and 3^rd^ stages, *E. amylovora* can infect flowers, with twigs and leaves in the 4^th^ and 5^th^ stages suggest that apple endosphere microbiota may be introduced from flowers and leave buds. Therefore, appropriate management during the formation of flowers and leave buds is crucial for controlling the *E. amylovora* infection.

### 3.4. GS11 and Lipomyces starkeyi negative correlation species against E. amylovora

We quantified the linear correlation between two variables (the fungal community and the fire blight pathogen) using the Pearson correlation coefficient (PCC) analysis. The PCC ranges from +1 to –1 where +1 represents a perfect positive linear correlation, 0 represents no linear correlation and –1 represents a perfect negative linear correlation. We analyzed fungi that had positive or negative correlations (FDR, *Padj* < 0.01) with the fire blight pathogen in the apple fungal microbiota, regardless of tissue and compartments (endosphere and episphere) (Figure S3). Nine and three fungal species were found to have a negative direct correlation with the fire blight pathogen in the apple endosphere and episphere, respectively (Figure 4A,B). An interesting finding was that while individual fungal species had a negative correlation with the fire blight pathogen, the relationship between fungal species was positive. Specifically, the fungi species GS11 and *Lipomyces starkeyi* were both found to have a negative correlation with fire blight pathogen and were commonly present in both the apple endosphere and episphere. *L. starkeyi* is a yeast type of fungi, a well-known species for the production of microbial lipids [27]. It has been established that various yeast exhibit plant-growth promotion effects with particular attention being paid to the Genus of *Lipomyces* as post-harvest disease biocontrol agents [28].

### 3.5. Certain species on fungi community guild response to fire blight disease

**Figure 5.**
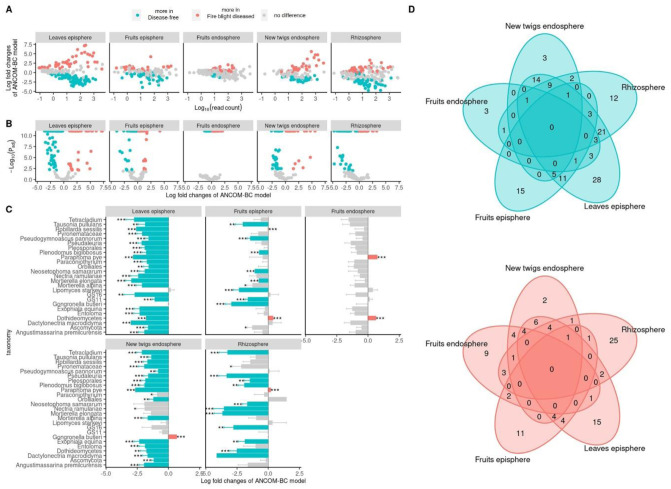
Distribution comparison of fungi using ANCOM-BC between disease-free and fire blight diseased trees at the 5th sampling time. The *P*-values were adjusted using the false discovery rate method in ANCOM-BC analysis. The cyan and pinky-red colors represent groups of fungi that are more abundant in disease-free and fire blight diseased trees, respectively (*P*_adj_ < 0.01). (A) and (B), *P*_adj_ values, log fold changes and read counts for each dot which corresponds to a fungal taxonomy group. (C), highlights ANCOM-BC results with a bar graph showing Erwiniaceae negatively correlated fungi in the network analysis (*: *P*_adj_ < 0.05, **: *P*_adj_ < 0.01, ***: *P*_adj_ < 0.001). Each panel is divided by tree organization. (D), the Venn diagram of differently distributed fungi. The cyan Venn diagram represents fungi that are more abundant in disease-free trees at the 5th sampling time while the pinky red Venn diagram represents fungi that are more abundant in fire blight diseased trees at the 5th sampling time.

The analysis of compositions of microbiomes with bias correction (ANCOM-BC) analyses was performed using the central log-ratio method to estimate absolute abundance from the relative abundance. A comparison of the apple tissue-specific fungi microbiota between disease-free and fire blight diseased orchards revealed changes in the abundance of various fungal species (Figure 5A,B). The fruit endosphere had the least number of changes in fungal abundance while the leaves episphere had the most changes in fungal abundance due to fire blight disease. A total of 25 fungal species were found to have a negative correlation with fire blight disease and ANCOM-BC analysis (*: *Padj* < 0.05, **: *Padj* < 0.01, ***: *Padj* < 0.001) results in at least one apple tissue (Fig. 5C). The results of predicting the ecological and phylogenetic guilds of 25 fungi associated with the fire blight pathogen and ANCOM-BC analysis revealed a negative correlation. These guilds were distributed as saprotroph (7 species), pathogen (4 species), endophyte (2 species), mycorrhiza (1 species), parasite (1 species) and unassigned (10 species) as shown in Table 2.

**Figure 6.**
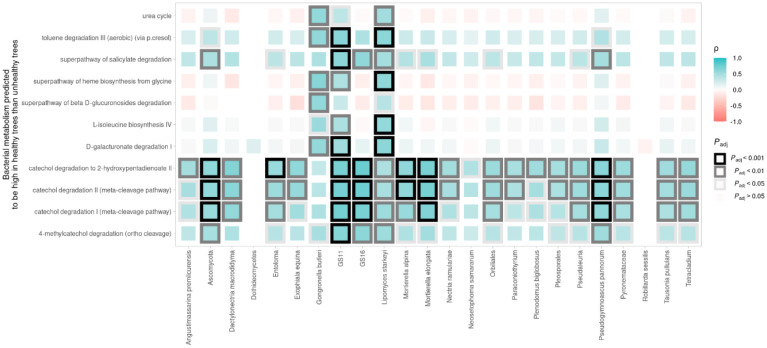
Correlation heatmap between bacterial metabolism and Erwiniaceae negatively correlated fungi in fire blight uninfected trees. Bacterial metabolism was predicted by PICRUSt2 and DESeq2 comparison between disease-free and fire blight diseased trees as reported in previous research. The Erwiniaceae negatively correlated fungi in the Pearson network analysis are arranged on the X-axis while the bacterial metabolism is arranged on the Y-axis. The colored squares at the intersection of the two axes represent the Pearson correlation ρ value and *P* value adjusted using the false discovery rate method. The filled and edge colors of the squares indicate the strength and direction of the correlation.

Kim et al. [22] observed that 11 microbiota metabolite pathways were significantly activated in the bacterial communities associated with disease-free apple trees in contrast to those associated with *E. amylovora*-infected apple trees. These 11 pathways can be grouped into four categories: nitrogen/carbon utilization, catechol degradation, salicylate degradation and toluene degradation. Notably, the correlation heatmap revealed a negative correlation between the previously reported metabolism of the bacterial community in disease-free apples and the fire blight pathogen. Furthermore, the fungal strain GS11 was found to exhibit significant metabolite activity in the catechol degradation pathway and the strain *Lipomyces starkeyi* was found to contribute to both nitrogen/carbon utilization and toluene degradation (Figure 6). These findings suggest that not only the bacterial community but also the fungi microbiome may play a significant role in maintaining the suppression of fire blight disease and maintaining balance in the holobiome communities in agricultural systems.

**Table 2.** FunGuild analysis results.

Taxonomy	Taxa level	Trophic mode	Guild	Growth form
Pleosporales	Order	-	-	-
*Pseudogymnoascus pannorum*	Species	Saprotroph	Plant Saprotroph-Wood Saprotroph	Microfungus
*Nectria ramulariae*	Species	Pathotroph-Saprotroph-Symbiotroph	Animal Pathogen-Endophyte-Fungal Parasite-Lichen Parasite-Plant Pathogen-Wood Saprotroph	Microfungus
Pyronemataceae	Family	Saprotroph-Symbiotroph	Dung Saprotroph-Ectomycorrhizal-Soil Saprotroph-Wood Saprotroph	Gasteroid-Pezizoid
*Pseudaleuria*	Genus	Saprotroph	Undefined Saprotroph	-
*Tausonia pullulans*	Species	-	-	-
*Mortierella alpi-*	Species	Saprotroph-Symbiotroph	Endophyte-Litter Saprotroph-Soil Saprotroph-Undefined Saprotroph	Microfungus
Ascomycota	Phylum	-	-	-
GS11	Order	-	-	-
*Paraconiothyrium*	Genus	Saprotroph	Undefined Saprotroph	-
*Lipomyces starkeyi*	Species	-	-	-
*Entoloma*	Genus	Pathotroph-Saprotroph-Symbiotroph	Ectomycorrhizal-Fungal Parasite-Soil Saprotroph-Undefined Saprotroph	Agaricoid
*Paraphoma pye*	Species	Pathotroph-Saprotroph	Fungal Parasite-Plant Pathogen-Plant Saprotroph	Microfungus
*Dactylonectria macrodidyma*	Species	Pathotroph-Saprotroph-Symbiotroph	Animal Pathogen-Endophyte-Fungal Parasite-Lichen Parasite-Plant Pathogen-Wood Saprotroph	Microfungus
Orbiliales	Order	-	-	-
*Tetracladium*	Genus	Saprotroph	Undefined Saprotroph	-
*Exophiala equi-*	Species	Pathotroph-Saprotroph	Animal Pathogen-Fungal Parasite-Undefined Saprotroph	Facultative Yeast-Microfungus
*Gongronella butleri*	Species	Saprotroph	Undefined Saprotroph	-
*Angustimassari-premilcurensis*	Species	-	-	-
*Mortierella elongata*	Species	Saprotroph-Symbiotroph	Endophyte-Litter Saprotroph-Soil Saprotroph-Undefined Saprotroph	Microfungus
GS16	Order	-	-	-
*Plenodomus biglobosus*	Species	Saprotroph	Undefined Saprotroph	-
*Neosetophoma samararum*	Species	Saprotroph	Undefined Saprotroph	-
*Robillarda sessilis*	Species	-	-	-
Dothideomycetes	Class	-	-	-

## 4. Conclusion

Apples are globally significant economic crops. Fire blight, one of the diseases that occur in apples, is a devastating disease that can cause the death of trees. In this study, an analysis of the microbiota was conducted to identify important fungi for the health of apples. Through in-depth analysis of the fungal microbiota in relation to the development of apples and the occurrence of fire blight disease, it was revealed that the strains GS11 and *Lipomyces starkeyi* are closely associated with the health of apples. Furthermore, it was found that the fungal microbiota in the rhizosphere of apple tissues is unrelated to the occurrence of fire blight disease. Although this study analytically examined the relationship between fungal microbiota and fire blight, it is suggested that future research should focus on disease management through the acquisition of microbial resources for the control of fire blight disease.
