# Supplementary material for: Mycobiota community and fungal species response to development stage and fire blight disease in apples
Source: AIMS Microbiol. 2023 Jul 20;9(3):554–69. doi: 10.3934/microbiol.2023029 (PMC10462452; doi:10.3934/microbiol.2023029)
Supplement: Supplementary file 1 [file microbiol-09-03-029-s001.pdf]

Microbiology

AIMS Microbiology, 9(3): 554–569.
DOI: 10.3934/microbiol.2023029
Received: 15 March 2023
Revised: 29 June 2023
Accepted: 12 July 2023
Published: 20 July 2023

http://www.aimspress.com/journal/microbiology

Research article

Mycobiota community and fungal species response to development stage and fire blight disease in apples

Su In Lee[1,†], Gyeongjun Cho[2,†], Su-Hyeon Kim[1], Da-Ran Kim[3], Youn-Sig Kwak[1,3,*]

[1] Division of Applied Life Science (BK21 Plus), Gyeongsang National University, Jinju 52828, Republic of Korea
[2] Division of Agricultural Microbiology, National Institute of Agriculture Science, Rural Development Administration, Wanju 55365, Republic of Korea
[3] Research Institute of Life Science, Gyeongsang National University, Jinju 52828, Republic of Korea

* **Correspondence:** Email: kwak@gnu.ac.kr; Tel: +82557721922: Fax: +82557721920.

† These two authors contributed equally.

Table S1. Pairwise PERMANOVA with beta diversity based on Bray-Curtis distance.

Compare1				Compare2						
Fireblight	Orgnization	Narrow region	Sampling time	Fireblight	Orgnization	Narrow region	Sampling time	P	FDR	
Healthy	Leaves	Endosphere	1st	Healthy	Rhizosphere	Rhizosphere	1st	0.006	0.012	*
Healthy	Leaves	Endosphere	1st	Healthy	Flowers and Fruits	Endosphere	2nd	0.033	0.037	*
Healthy	Leaves	Endosphere	1st	Healthy	Rhizosphere	Rhizosphere	2nd	0.007	0.013	*
Healthy	Leaves	Endosphere	1st	Healthy	Flowers and Fruits	Endosphere	3rd	0.01	0.014	*
Healthy	Leaves	Endosphere	1st	Healthy	Rhizosphere	Rhizosphere	3rd	0.007	0.013	*
Healthy	Leaves	Endosphere	1st	Healthy	New twigs	Endosphere	4th	0.007	0.013	*
Healthy	Leaves	Endosphere	1st	Healthy	Rhizosphere	Rhizosphere	4th	0.02	0.024	*
Healthy	Leaves	Endosphere	1st	Healthy	New twigs	Endosphere	5th	0.01	0.014	*
Healthy	Leaves	Endosphere	1st	Healthy	Flowers and Fruits	Endosphere	5th	0.01	0.014	*
Healthy	Leaves	Endosphere	1st	Healthy	Flowers and Fruits	Episphere	5th	0.007	0.013	*
Healthy	Leaves	Endosphere	1st	Healthy	Leaves	Episphere	5th	0.012	0.016	*
Healthy	Leaves	Endosphere	1st	Healthy	Rhizosphere	Rhizosphere	5th	0.004	0.009	**
Healthy	Leaves	Endosphere	1st	Unhealthy	New twigs	Endosphere	5th	0.001	0.004	**
Healthy	Leaves	Endosphere	1st	Unhealthy	Flowers and Fruits	Endosphere	5th	0.032	0.037	*
Healthy	Leaves	Endosphere	1st	Unhealthy	Flowers and Fruits	Episphere	5th	0.001	0.004	**
Healthy	Leaves	Endosphere	1st	Unhealthy	Leaves	Episphere	5th	0.001	0.004	**
Healthy	Leaves	Endosphere	1st	Unhealthy	Rhizosphere	Rhizosphere	5th	0.004	0.009	**
Healthy	Rhizosphere	Rhizosphere	1st	Healthy	Flowers and Fruits	Endosphere	2nd	0.012	0.016	*
Healthy	Rhizosphere	Rhizosphere	1st	Healthy	Rhizosphere	Rhizosphere	2nd	0.601	0.605	no significant
Healthy	Rhizosphere	Rhizosphere	1st	Healthy	Flowers and Fruits	Endosphere	3rd	0.008	0.013	*
Healthy	Rhizosphere	Rhizosphere	1st	Healthy	Rhizosphere	Rhizosphere	3rd	0.096	0.105	no significant
Healthy	Rhizosphere	Rhizosphere	1st	Healthy	New twigs	Endosphere	4th	0.008	0.013	*
Healthy	Rhizosphere	Rhizosphere	1st	Healthy	Rhizosphere	Rhizosphere	4th	0.025	0.03	*
Healthy	Rhizosphere	Rhizosphere	1st	Healthy	New twigs	Endosphere	5th	0.008	0.013	*
Healthy	Rhizosphere	Rhizosphere	1st	Healthy	Flowers and Fruits	Endosphere	5th	0.01	0.014	*
Healthy	Rhizosphere	Rhizosphere	1st	Healthy	Flowers and Fruits	Episphere	5th	0.01	0.014	*
Healthy	Rhizosphere	Rhizosphere	1st	Healthy	Leaves	Episphere	5th	0.009	0.014	*
Healthy	Rhizosphere	Rhizosphere	1st	Healthy	Rhizosphere	Rhizosphere	5th	0.226	0.238	no significant
Healthy	Rhizosphere	Rhizosphere	1st	Unhealthy	New twigs	Endosphere	5th	0.002	0.005	**
Healthy	Rhizosphere	Rhizosphere	1st	Unhealthy	Flowers and Fruits	Endosphere	5th	0.003	0.007	**
Healthy	Rhizosphere	Rhizosphere	1st	Unhealthy	Flowers and Fruits	Episphere	5th	0.001	0.004	**
Healthy	Rhizosphere	Rhizosphere	1st	Unhealthy	Leaves	Episphere	5th	0.001	0.004	**
Healthy	Rhizosphere	Rhizosphere	1st	Unhealthy	Rhizosphere	Rhizosphere	5th	0.008	0.013	*
Healthy	Flowers and Fruits	Endosphere	2nd	Healthy	Rhizosphere	Rhizosphere	2nd	0.023	0.027	*
Healthy	Flowers and Fruits	Endosphere	2nd	Healthy	Flowers and Fruits	Endosphere	3rd	0.032	0.037	*
Healthy	Flowers and Fruits	Endosphere	2nd	Healthy	Rhizosphere	Rhizosphere	3rd	0.011	0.015	*

Continued on next page

Compare1				Compare2						
Fireblight	Orgnization	Narrow region	Sampling time	Fireblight	Orgnization	Narrow region	Sampling time	P	FDR	
Healthy	Flowers and Fruits	Endosphere	2nd	Healthy	New twigs	Endosphere	4th	0.027	0.032	*
Healthy	Flowers and Fruits	Endosphere	2nd	Healthy	Rhizosphere	Rhizosphere	4th	0.164	0.175	no significant
Healthy	Flowers and Fruits	Endosphere	2nd	Healthy	New twigs	Endosphere	5th	0.016	0.02	*
Healthy	Flowers and Fruits	Endosphere	2nd	Healthy	Flowers and Fruits	Endosphere	5th	0.009	0.014	*
Healthy	Flowers and Fruits	Endosphere	2nd	Healthy	Flowers and Fruits	Episphere	5th	0.007	0.013	*
Healthy	Flowers and Fruits	Endosphere	2nd	Healthy	Leaves	Episphere	5th	0.043	0.048	*
Healthy	Flowers and Fruits	Endosphere	2nd	Healthy	Rhizosphere	Rhizosphere	5th	0.008	0.013	*
Healthy	Flowers and Fruits	Endosphere	2nd	Unhealthy	New twigs	Endosphere	5th	0.001	0.004	**
Healthy	Flowers and Fruits	Endosphere	2nd	Unhealthy	Flowers and Fruits	Endosphere	5th	0.006	0.012	*
Healthy	Flowers and Fruits	Endosphere	2nd	Unhealthy	Flowers and Fruits	Episphere	5th	0.001	0.004	**
Healthy	Flowers and Fruits	Endosphere	2nd	Unhealthy	Leaves	Episphere	5th	0.001	0.004	**
Healthy	Flowers and Fruits	Endosphere	2nd	Unhealthy	Rhizosphere	Rhizosphere	5th	0.001	0.004	**
Healthy	Rhizosphere	Rhizosphere	2nd	Healthy	Flowers and Fruits	Endosphere	3rd	0.005	0.011	*
Healthy	Rhizosphere	Rhizosphere	2nd	Healthy	Rhizosphere	Rhizosphere	3rd	0.563	0.57	no significant
Healthy	Rhizosphere	Rhizosphere	2nd	Healthy	New twigs	Endosphere	4th	0.005	0.011	*
Healthy	Rhizosphere	Rhizosphere	2nd	Healthy	Rhizosphere	Rhizosphere	4th	0.103	0.112	no significant
Healthy	Rhizosphere	Rhizosphere	2nd	Healthy	New twigs	Endosphere	5th	0.005	0.011	*
Healthy	Rhizosphere	Rhizosphere	2nd	Healthy	Flowers and Fruits	Endosphere	5th	0.009	0.014	*
Healthy	Rhizosphere	Rhizosphere	2nd	Healthy	Flowers and Fruits	Episphere	5th	0.006	0.012	*
Healthy	Rhizosphere	Rhizosphere	2nd	Healthy	Leaves	Episphere	5th	0.011	0.015	*
Healthy	Rhizosphere	Rhizosphere	2nd	Healthy	Rhizosphere	Rhizosphere	5th	0.418	0.426	no significant
Healthy	Rhizosphere	Rhizosphere	2nd	Unhealthy	New twigs	Endosphere	5th	0.001	0.004	**
Healthy	Rhizosphere	Rhizosphere	2nd	Unhealthy	Flowers and Fruits	Endosphere	5th	0.001	0.004	**
Healthy	Rhizosphere	Rhizosphere	2nd	Unhealthy	Flowers and Fruits	Episphere	5th	0.001	0.004	**
Healthy	Rhizosphere	Rhizosphere	2nd	Unhealthy	Leaves	Episphere	5th	0.001	0.004	**
Healthy	Rhizosphere	Rhizosphere	2nd	Unhealthy	Rhizosphere	Rhizosphere	5th	0.008	0.013	*
Healthy	Flowers and Fruits	Endosphere	3rd	Healthy	Rhizosphere	Rhizosphere	3rd	0.014	0.017	*
Healthy	Flowers and Fruits	Endosphere	3rd	Healthy	New twigs	Endosphere	4th	0.119	0.128	no significant
Healthy	Flowers and Fruits	Endosphere	3rd	Healthy	Rhizosphere	Rhizosphere	4th	0.01	0.014	*
Healthy	Flowers and Fruits	Endosphere	3rd	Healthy	New twigs	Endosphere	5th	0.033	0.037	*
Healthy	Flowers and Fruits	Endosphere	3rd	Healthy	Flowers and Fruits	Endosphere	5th	0.006	0.012	*
Healthy	Flowers and Fruits	Endosphere	3rd	Healthy	Flowers and Fruits	Episphere	5th	0.006	0.012	*
Healthy	Flowers and Fruits	Endosphere	3rd	Healthy	Leaves	Episphere	5th	0.035	0.039	*
Healthy	Flowers and Fruits	Endosphere	3rd	Healthy	Rhizosphere	Rhizosphere	5th	0.01	0.014	*
Healthy	Flowers and Fruits	Endosphere	3rd	Unhealthy	New twigs	Endosphere	5th	0.002	0.005	**
Healthy	Flowers and Fruits	Endosphere	3rd	Unhealthy	Flowers and Fruits	Endosphere	5th	0.001	0.004	**
Healthy	Flowers and Fruits	Endosphere	3rd	Unhealthy	Flowers and Fruits	Episphere	5th	0.001	0.004	**
Healthy	Flowers and Fruits	Endosphere	3rd	Unhealthy	Leaves	Episphere	5th	0.001	0.004	**
Healthy	Flowers and Fruits	Endosphere	3rd	Unhealthy	Rhizosphere	Rhizosphere	5th	0.001	0.004	**

Continued on next page

Compare1				Compare2						
Fireblight	Orgnization	Narrow region	Sampling time	Fireblight	Orgnization	Narrow region	Sampling time	P	FDR	
Healthy	Rhizosphere	Rhizosphere	3rd	Healthy	New twigs	Endosphere	4th	0.008	0.013	*
Healthy	Rhizosphere	Rhizosphere	3rd	Healthy	Rhizosphere	Rhizosphere	4th	0.341	0.355	no significant
Healthy	Rhizosphere	Rhizosphere	3rd	Healthy	New twigs	Endosphere	5th	0.008	0.013	*
Healthy	Rhizosphere	Rhizosphere	3rd	Healthy	Flowers and Fruits	Endosphere	5th	0.008	0.013	*
Healthy	Rhizosphere	Rhizosphere	3rd	Healthy	Flowers and Fruits	Episphere	5th	0.008	0.013	*
Healthy	Rhizosphere	Rhizosphere	3rd	Healthy	Leaves	Episphere	5th	0.01	0.014	*
Healthy	Rhizosphere	Rhizosphere	3rd	Healthy	Rhizosphere	Rhizosphere	5th	0.865	0.865	no significant
Healthy	Rhizosphere	Rhizosphere	3rd	Unhealthy	New twigs	Endosphere	5th	0.001	0.004	**
Healthy	Rhizosphere	Rhizosphere	3rd	Unhealthy	Flowers and Fruits	Endosphere	5th	0.001	0.004	**
Healthy	Rhizosphere	Rhizosphere	3rd	Unhealthy	Flowers and Fruits	Episphere	5th	0.001	0.004	**
Healthy	Rhizosphere	Rhizosphere	3rd	Unhealthy	Leaves	Episphere	5th	0.004	0.009	**
Healthy	Rhizosphere	Rhizosphere	3rd	Unhealthy	Rhizosphere	Rhizosphere	5th	0.002	0.005	**
Healthy	New twigs	Endosphere	4th	Healthy	Rhizosphere	Rhizosphere	4th	0.012	0.016	*
Healthy	New twigs	Endosphere	4th	Healthy	New twigs	Endosphere	5th	0.08	0.088	no significant
Healthy	New twigs	Endosphere	4th	Healthy	Flowers and Fruits	Endosphere	5th	0.017	0.021	*
Healthy	New twigs	Endosphere	4th	Healthy	Flowers and Fruits	Episphere	5th	0.013	0.016	*
Healthy	New twigs	Endosphere	4th	Healthy	Leaves	Episphere	5th	0.027	0.032	*
Healthy	New twigs	Endosphere	4th	Healthy	Rhizosphere	Rhizosphere	5th	0.013	0.016	*
Healthy	New twigs	Endosphere	4th	Unhealthy	New twigs	Endosphere	5th	0.001	0.004	**
Healthy	New twigs	Endosphere	4th	Unhealthy	Flowers and Fruits	Endosphere	5th	0.002	0.005	**
Healthy	New twigs	Endosphere	4th	Unhealthy	Flowers and Fruits	Episphere	5th	0.001	0.004	**
Healthy	New twigs	Endosphere	4th	Unhealthy	Leaves	Episphere	5th	0.001	0.004	**
Healthy	New twigs	Endosphere	4th	Unhealthy	Rhizosphere	Rhizosphere	5th	0.001	0.004	**
Healthy	Rhizosphere	Rhizosphere	4th	Healthy	New twigs	Endosphere	5th	0.005	0.011	*
Healthy	Rhizosphere	Rhizosphere	4th	Healthy	Flowers and Fruits	Endosphere	5th	0.007	0.013	*
Healthy	Rhizosphere	Rhizosphere	4th	Healthy	Flowers and Fruits	Episphere	5th	0.01	0.014	*
Healthy	Rhizosphere	Rhizosphere	4th	Healthy	Leaves	Episphere	5th	0.273	0.286	no significant
Healthy	Rhizosphere	Rhizosphere	4th	Healthy	Rhizosphere	Rhizosphere	5th	0.391	0.401	no significant
Healthy	Rhizosphere	Rhizosphere	4th	Unhealthy	New twigs	Endosphere	5th	0.001	0.004	**
Healthy	Rhizosphere	Rhizosphere	4th	Unhealthy	Flowers and Fruits	Endosphere	5th	0.003	0.007	**
Healthy	Rhizosphere	Rhizosphere	4th	Unhealthy	Flowers and Fruits	Episphere	5th	0.001	0.004	**
Healthy	Rhizosphere	Rhizosphere	4th	Unhealthy	Leaves	Episphere	5th	0.001	0.004	**
Healthy	Rhizosphere	Rhizosphere	4th	Unhealthy	Rhizosphere	Rhizosphere	5th	0.007	0.013	*
Healthy	New twigs	Endosphere	5th	Healthy	Flowers and Fruits	Endosphere	5th	0.017	0.021	*
Healthy	New twigs	Endosphere	5th	Healthy	Flowers and Fruits	Episphere	5th	0.007	0.013	*
Healthy	New twigs	Endosphere	5th	Healthy	Leaves	Episphere	5th	0.371	0.384	no significant
Healthy	New twigs	Endosphere	5th	Healthy	Rhizosphere	Rhizosphere	5th	0.008	0.013	*
Healthy	New twigs	Endosphere	5th	Unhealthy	New twigs	Endosphere	5th	0.002	0.005	**
Healthy	New twigs	Endosphere	5th	Unhealthy	Flowers and Fruits	Endosphere	5th	0.002	0.005	**

Continued on next page

Compare1				Compare2						
Fireblight	Orgnization	Narrow region	Sampling time	Fireblight	Orgnization	Narrow region	Sampling time	P	FDR	
Healthy	New twigs	Endosphere	5th	Unhealthy	Flowers and Fruits	Episphere	5th	0.001	0.004	**
Healthy	New twigs	Endosphere	5th	Unhealthy	Leaves	Episphere	5th	0.002	0.005	**
Healthy	New twigs	Endosphere	5th	Unhealthy	Rhizosphere	Rhizosphere	5th	0.002	0.005	**
Healthy	Flowers and Fruits	Endosphere	5th	Healthy	Flowers and Fruits	Episphere	5th	0.01	0.014	*
Healthy	Flowers and Fruits	Endosphere	5th	Healthy	Leaves	Episphere	5th	0.011	0.015	*
Healthy	Flowers and Fruits	Endosphere	5th	Healthy	Rhizosphere	Rhizosphere	5th	0.014	0.017	*
Healthy	Flowers and Fruits	Endosphere	5th	Unhealthy	New twigs	Endosphere	5th	0.001	0.004	**
Healthy	Flowers and Fruits	Endosphere	5th	Unhealthy	Flowers and Fruits	Endosphere	5th	0.013	0.016	*
Healthy	Flowers and Fruits	Endosphere	5th	Unhealthy	Flowers and Fruits	Episphere	5th	0.001	0.004	**
Healthy	Flowers and Fruits	Endosphere	5th	Unhealthy	Leaves	Episphere	5th	0.001	0.004	**
Healthy	Flowers and Fruits	Endosphere	5th	Unhealthy	Rhizosphere	Rhizosphere	5th	0.002	0.005	**
Healthy	Flowers and Fruits	Episphere	5th	Healthy	Leaves	Episphere	5th	0.01	0.014	*
Healthy	Flowers and Fruits	Episphere	5th	Healthy	Rhizosphere	Rhizosphere	5th	0.008	0.013	*
Healthy	Flowers and Fruits	Episphere	5th	Unhealthy	New twigs	Endosphere	5th	0.002	0.005	**
Healthy	Flowers and Fruits	Episphere	5th	Unhealthy	Flowers and Fruits	Endosphere	5th	0.003	0.007	**
Healthy	Flowers and Fruits	Episphere	5th	Unhealthy	Flowers and Fruits	Episphere	5th	0.001	0.004	**
Healthy	Flowers and Fruits	Episphere	5th	Unhealthy	Leaves	Episphere	5th	0.001	0.004	**
Healthy	Flowers and Fruits	Episphere	5th	Unhealthy	Rhizosphere	Rhizosphere	5th	0.001	0.004	**
Healthy	Leaves	Episphere	5th	Healthy	Rhizosphere	Rhizosphere	5th	0.032	0.037	*
Healthy	Leaves	Episphere	5th	Unhealthy	New twigs	Endosphere	5th	0.001	0.004	**
Healthy	Leaves	Episphere	5th	Unhealthy	Flowers and Fruits	Endosphere	5th	0.003	0.007	**
Healthy	Leaves	Episphere	5th	Unhealthy	Flowers and Fruits	Episphere	5th	0.002	0.005	**
Healthy	Leaves	Episphere	5th	Unhealthy	Leaves	Episphere	5th	0.002	0.005	**
Healthy	Leaves	Episphere	5th	Unhealthy	Rhizosphere	Rhizosphere	5th	0.001	0.004	**
Healthy	Rhizosphere	Rhizosphere	5th	Unhealthy	New twigs	Endosphere	5th	0.002	0.005	**
Healthy	Rhizosphere	Rhizosphere	5th	Unhealthy	Flowers and Fruits	Endosphere	5th	0.001	0.004	**
Healthy	Rhizosphere	Rhizosphere	5th	Unhealthy	Flowers and Fruits	Episphere	5th	0.002	0.005	**
Healthy	Rhizosphere	Rhizosphere	5th	Unhealthy	Leaves	Episphere	5th	0.001	0.004	**
Healthy	Rhizosphere	Rhizosphere	5th	Unhealthy	Rhizosphere	Rhizosphere	5th	0.011	0.015	*
Unhealthy	New twigs	Endosphere	5th	Unhealthy	Flowers and Fruits	Endosphere	5th	0.183	0.194	no significant
Unhealthy	New twigs	Endosphere	5th	Unhealthy	Flowers and Fruits	Episphere	5th	0.004	0.009	**
Unhealthy	New twigs	Endosphere	5th	Unhealthy	Leaves	Episphere	5th	0.004	0.009	**
Unhealthy	New twigs	Endosphere	5th	Unhealthy	Rhizosphere	Rhizosphere	5th	0.001	0.004	**
Unhealthy	Flowers and Fruits	Endosphere	5th	Unhealthy	Flowers and Fruits	Episphere	5th	0.007	0.013	*
Unhealthy	Flowers and Fruits	Endosphere	5th	Unhealthy	Leaves	Episphere	5th	0.002	0.005	**
Unhealthy	Flowers and Fruits	Endosphere	5th	Unhealthy	Rhizosphere	Rhizosphere	5th	0.002	0.005	**
Unhealthy	Flowers and Fruits	Episphere	5th	Unhealthy	Leaves	Episphere	5th	0.001	0.004	**
Unhealthy	Flowers and Fruits	Episphere	5th	Unhealthy	Rhizosphere	Rhizosphere	5th	0.001	0.004	**
Unhealthy	Leaves	Episphere	5th	Unhealthy	Rhizosphere	Rhizosphere	5th	0.001	0.004	**

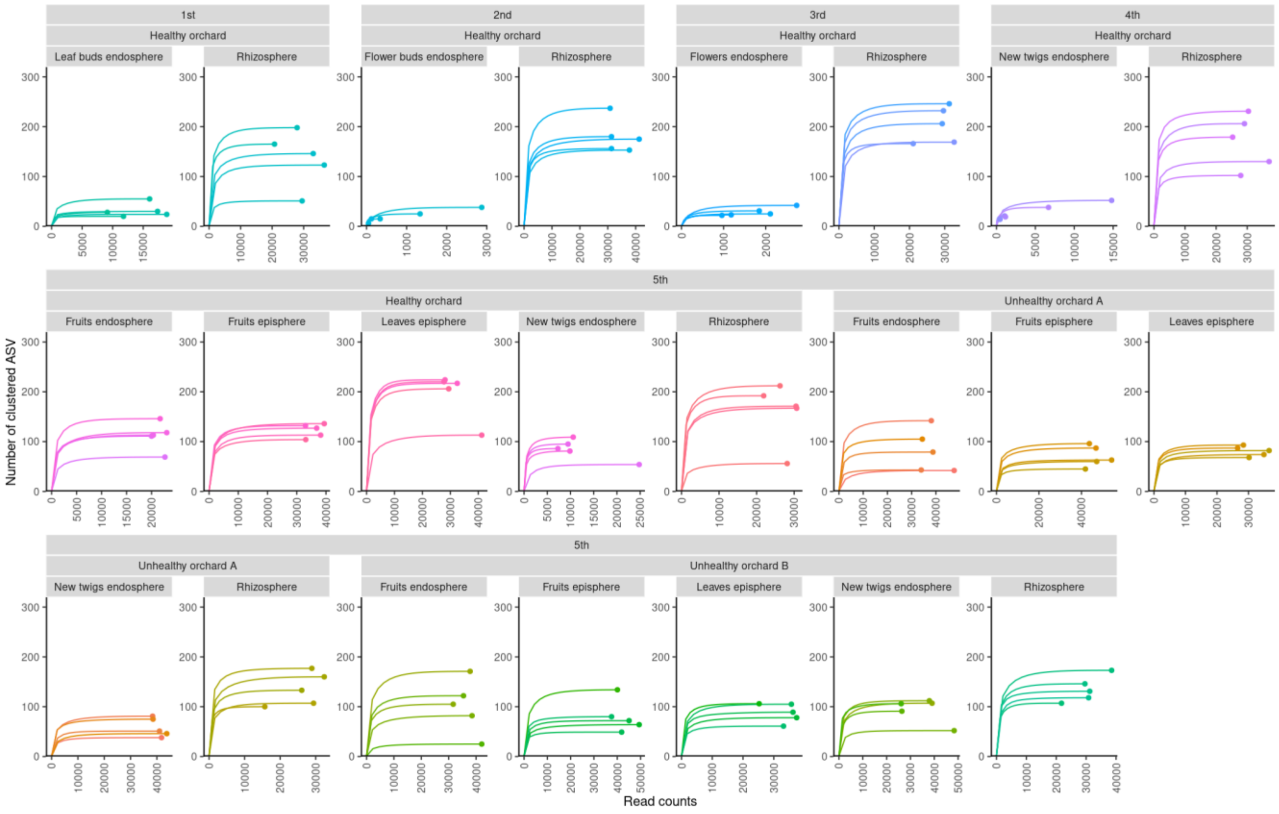

Figure S1. Rarefaction curve generated from the ITS2 region library using ASV. The X-axis represents the read counts, while the Y-axis represents the clustered ASV. Each curve in the small panel represents the same condition sample described in the upper strip.

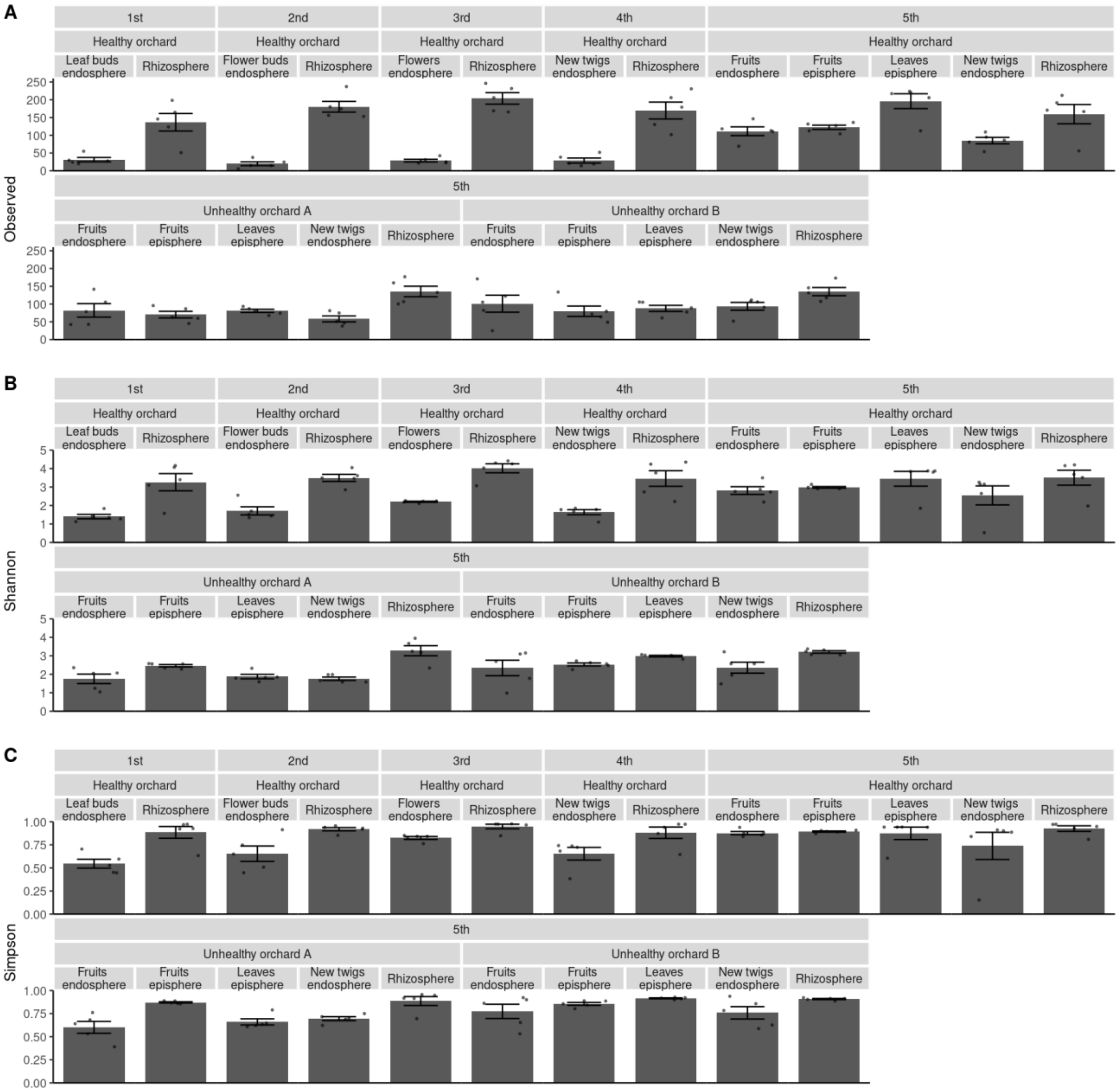

Figure S2. Alpha diversity of all samples. (A) observed ASV. (B) Shannon index. (C) Simpson index.

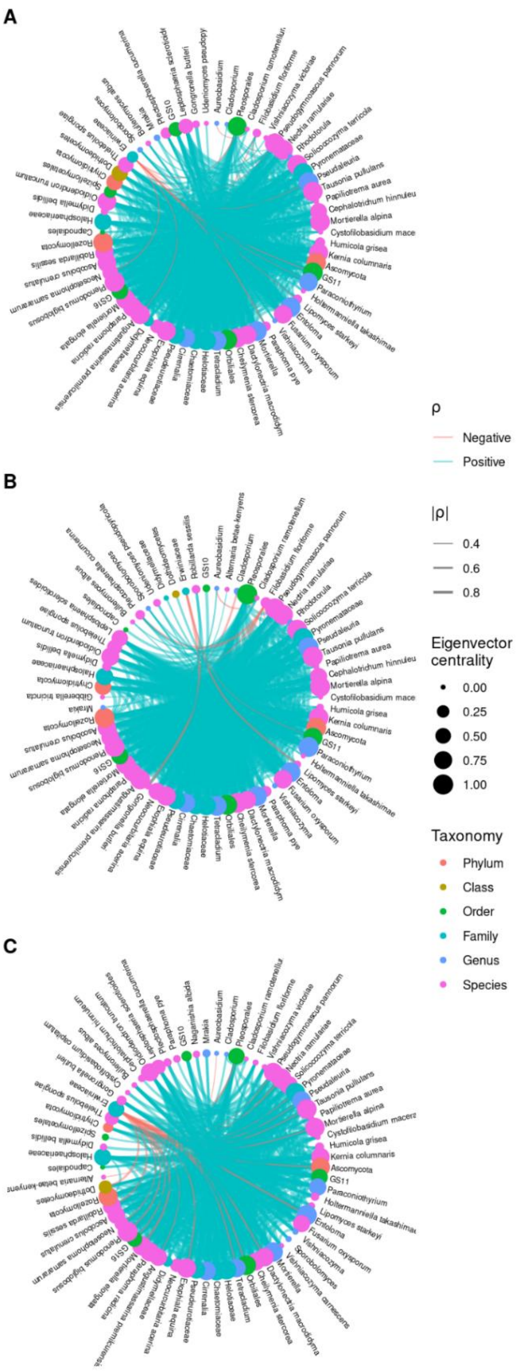

Figure S3. Pearson correlation network of fungi and Erwiniaceae in the endosphere and episphere. (A) Endosphere and episphere combined, (B) Endosphere, (C) Episphere. False discovery rate (FDR) was used for adjusting the analysis, and all interactions among Erwiniaceae-correlated fungi with a P_{adj} value <0.01 are displayed. Nodes represent different taxonomy groups, distinguished by color and eigenvector centrality, indicating taxonomy level and centrality. The thickness and color of lines indicate correlation strength (rho values).

AIMS Press

©2023 the Author(s), licensee AIMS Press. This is an open access article distributed under the terms of the Creative Commons Attribution License (http://creativecommons.org/licenses/by/4.0)
